# Mechanism of melanoma cells selective apoptosis induced by a photoactive NADPH analogue

**DOI:** 10.18632/oncotarget.12651

**Published:** 2016-10-14

**Authors:** Florian Rouaud, Jean-Luc Boucher, Anny Slama-Schwok, Stéphane Rocchi

**Affiliations:** ^1^ INSERM U1065 Team 1, Université de Nice Sophia Antipolis et Centre Méditerranéen de Médecine Moléculaire, Nice, France; ^2^ CNRS UMR 8601, Université Paris Descartes, Paris, France; ^3^ Paris Saclay University, UR 892 INRA, Jouy en Josas, France

**Keywords:** melanoma, NADPH oxidase, ROS, ER stress, NADPH analogue

## Abstract

Melanoma is one of the most lethal cancers when it reaches a metastatic stage. Despite the spectacular achievements of targeted therapies (BRAF inhibitors) or immuno-therapies (anti-CTLA4 or anti-PD1), most patients with melanoma will need additional treatments. Here we used a photoactive NADPH analogue called NS1 to induce cell death by inhibition of NADPH oxidases NOX in melanoma cells, including melanoma cells isolated from patients. In contrast, healthy melanocytes growth was unaffected by NS1 treatment.

NS1 established an early Endoplasmic Reticulum stress by the early release of calcium mediated by (a) calcium-dependent redox-sensitive ion channel(s). These events initiated autophagy and apoptosis in all tested melanoma cells independently of their mutational status. The autophagy promoted by NS1 was incomplete. The autophagic flux was blocked at late stage events, consistent with the accumulation of p62, and a close localization of LC3 with NS1 associated with NS1 inhibition of NOX1 in autophagosomes. This hypothesis of a specific incomplete autophagy and apoptosis driven by NS1 was comforted by the use of siRNAs and pharmacological inhibitors blocking different processes. This study highlights the potential therapeutic interest of NS1 inducing cell death by triggering a selective ER stress and incomplete autophagy in melanoma cells harbouring wt and BRAF mutation.

## INTRODUCTION

Cutaneous melanoma deriving from the transformation of melanocytes is one of the most lethal cancers among young adults. Melanoma has a high capability of invasion and rapidly metastasizes to other organs (lymph node, lung, liver, brain…). Patients with metastatic melanoma have short lifetime expectancy. Indeed, at the stage of visceral metastasis, the prognosis is catastrophic with a median survival of 6 months. Therefore, although melanoma represents only 5 % of all cutaneous cancers, it is responsible for 80 % of the deaths associated with skin cancers [1].

Encouraging results have recently been obtained with BRAF inhibitors, Vemurafenib (PLX4032) or Dabrafenib, and with MEK inhibitors [2]. While these inhibitors [3–5] increase the lifetime expectancy of patients by about 6 months, regrettably, after a short period of remission, melanomas acquire drug resistance. Recurrence of metastases is mostly observed. Other therapies recently developed to reactivate the immune response of the patient (anti-CTLA4 and anti-PD1) [6, 7] result in an objective and long-lasting response in about 15 to 35 % of patients.

Melanoma cells are characterized by altered redox signalling, in particular higher Radical Oxygen Species (ROS) levels than required for normal cell signalling [8]. Overexpressed levels of two isoforms of NADPH oxidase (NOX) are active in producing high ROS levels in melanoma: NOX4 and NOX1. NOX4 has been involved in cell survival and angiogenesis [9, 10] while NOX1 was shown to participate in EMT [11]. NO produced by the NO-synthases may act as a pro-angiogenic factor in many cancers. Both endothelial and neuronal NO-synthases have been linked with metastasis [12] and modulation of several signaling pathways active in melanoma as Notch and Interferon [13]. Melanoma-cell derived NO is a crucial modulator of immune function in the tumor microenvironment and provides a potentially novel target for treatment [14]. ROS formed by uncoupled eNOS and toxic levels of NO formed by iNOS participate in melanoma progression [15, 16]. To counteract the potential damaging effects of high ROS/NO concentrations (DNA damage, mutations, induction of apoptosis), melanoma cells have acquired efficient antioxidant strategies, based on overexpressed catalase and SOD imbedded in the membrane close to NOX1 [17], the remaining ROS (after catalase and SOD action) has a stimulating effect for proliferation of tumor cells.

Inhibition of ROS formed by NADPH oxidases (NOX) and/or by eNOS uncoupling is highly requested for pharmacological treatments of oxidative stress associated with cancers [18]. A rational way to regulate redox stress would be to make use of compounds modulating NADPH levels, which requires selectivity toward specific NADPH-dependent enzymes without strong interference with normal cellular processes. Recently, we designed a novel photoactive probe, called nanoshutter (NS1) that efficiently bound to constitutive NOS (eNOS and nNOS) [19] in a similar manner than our previously reported dienic nanotrigger [20–23]. NS1 reversibly inhibited NO formed by recombinant eNOS in endothelial cells by competing with NADPH binding and presented anti-angiogenic effects [19]. NS1 specifically inhibited melanoma cell growth [24]. This process was dependent upon NS1 inducing a decrease of ROS formed in A375 cells.

In the present manuscript, we explore the mechanism of NS1-induced inhibition of melanoma cell growth. In multiple metastatic melanoma cells, NS1 induced a stress of the endoplasmic reticulum associated with an early calcium release. The stress triggered incomplete autophagy by inhibition of ROS in all tested melanoma cells. Independent measurements confirmed that bound NS1 inhibited NOX.

## RESULTS

### Effect of NS1 on metastatic melanoma cells survival and ROS and NOX levels

**Figure 1:**
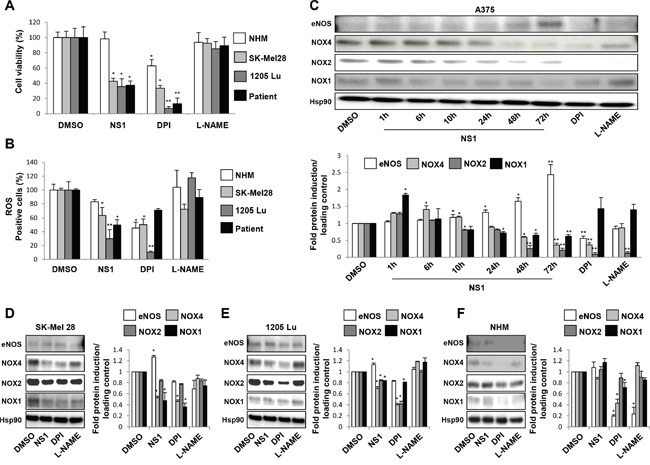
NS1-induced changes in melanoma cells and melanocytes viability and in eNOS and NOX isoforms **A.** Different melanoma cells or normal melanocytes were treated by 30μM NS1 or 30μM DPI or 100 μM L-NAME during 72h. The cell viability in A375 and other melanoma cells was determined by trypan blue staining. **B.** the percentage of ROS formed in the various melanoma cells and melanocytes (NHM) was analyzed by the measurement of fluorescence of the CellROX Deep Red reagent. DMSO: control buffer. The results are expressed as percentages of the control. **C-F.** Kinetics of eNOS, NOX1 and NOX4 levels in A375 melanoma cells (C) or SK-Mel 28 (D) and 1205 Lu melanoma cells (E) and melanocytes (F) were exposed at indicated times with 30μM NS1 or 30μM DPI or 100 μM L-NAME. Lysates were analyzed by western blot using the indicated antibodies. HSP90 was used as a loading control. One representative experiment out of three is shown. Data are quantified as mean ± SD of three independent experiments performed in triplicate. *, P < 0.05; **, P < 0.01; ***, P < 0.001.

NS1 decreased melanoma A375 cell viability [24]. Here we compare the effects of NS1 treatment on various melanoma cells and normal melanocytes. NS1 (30μM) strongly reduced the viability of multiple melanoma cells, SK-Mel28, which carried mutations of p53 and of BRAF, 1205 Lu cells mutated in BRAF and PTEN and melanoma cells freshly isolated from a patient at 72h without affecting the viability of healthy melanocytes (NHM) (Figure 1A).

Melanoma cells are characterized by an altered redox status, in particular higher ROS levels than required for normal cell signalling [25]. ROS levels were quantified by the CellRox reagent. A significant decrease of ROS was observed in all tested melanoma cells, reaching 70% inhibition of ROS levels in 1205 Lu cells at 72h (Figure 1B). This inhibition was also obtained with DPI inhibiting flavoenzymes (30μM). 1205 Lu cells were more sensitive than other melanoma cell lines to ROS inhibition and DPI inhibited ROS levels to a larger extent than NS1 did. In contrast, L-NAME (100μM) only slightly lowered ROS levels. The decrease of ROS levels at 72 hours was concomitant with a strong reduction of melanoma cell viability (Figure 1A and 1B).

**Figure 2:**
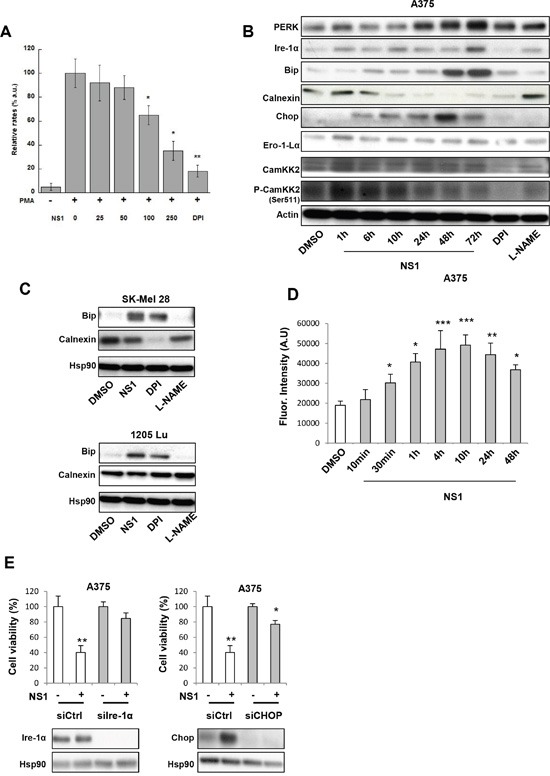
NS1 induces an early ER stress, modulated ROS levels and enhanced a release of calcium **A.** NS1 inhibits the formation of superoxide O_2_^−.^ in RAW 264.7 cells. Data compare the relative changes in the rates of formation of the DEPMPO-OOH spin adduct measured as the differences in the intensity of the first line of the DEPMPO-OOH spin-adduct and normalized to radical levels found in PMA-stimulated macrophages in the absence of NS1. Conditions are described in Experimental procedures and data are taken from 3-4 representative experiments. **B.** and **C.** Kinetics of ER stress markers expression in A375 melanoma cells (B) or other melanoma cells (C) were treated by 30μM NS1 or 30μM DPI or 100 μM L-NAME at indicated times. Cells were lysed and analyzed by western blotting using the indicated antibodies. HSP90 or actin was used as a loading control. One representative experiment out of three is shown. **D.** In parallel, calcium release was determined by monitoring the fluorescence of a calcium probe. The results are expressed as fluorescence intensity. Data are mean ± SD of three independent experiments performed in triplicate. **E.** A375 melanoma cells were transfected with a siRNA against Ire-1α (si Ire-1α), CHOP (siCHOP), or a siRNA control (siCtrl). Twenty-four hours after transfection, cells were treated with 30μM NS1 or DMSO for 72 hours. The results are expressed as percentages of the control. In parallel, cells were lysed and analyzed by western blotting using the indicated antibodies. HSP90 was used as a loading control. The results are expressed as percentages of the control.

NADPH oxidases are one of the main enzymatic sources of ROS in cells and the relative expression of NOX_1_ and NOX_4_was linked to melanoma cell survival [10, 26]. To identify which isoform(s) contributed to ROS levels in A375 cells, we tested the kinetics of eNOS, NOX1, NOX2, NOX4 expression as a function of time by western blots (Figure 1C). After 1 hour in the presence of NS1, a transient NOX1 increase was observed, which decreased after 24 hours and beyond. NOX2 and NOX4 strongly decreased, reaching a very low level after 72 hours. In agreement with a cross-talk between eNOS and NOX4, eNOS level increased [24]. NOX1 and NOX4 also decreased in SK-Mel28 and 1205 Lu cell lines as in A375 but smaller effects were observed in 1205 Lu. Only small changes in eNOS levels were observed in these cells as compared to the large increase observed in A375 cells (Figure 1C–1E). Since NOX4 is a self-sufficient enzyme, a decrease of NOX4 level directly reflects the ROS levels. Previous studies also showed that NS1 inhibits e/nNOS and NOX4 [19, 24]. The observed changes in NOX1 and NOX2 levels could not be directly linked with their ROS production since both proteins require additional proteins for full activation. To further confirm a direct inhibition of NOX by NS1, we used RAW 264.7 macrophage cells treated with PMA. PMA- activated NOX2- released superoxide anion radicals that were trapped by the cyclic nitrone DEPMPO. The nitroxide DEPMPO-OOH spin-adduct was detected by EPR spectroscopy. The addition of increasing amounts of NS1 to the PMA-stimulated RAW cells led to half-inhibition of the rates of DEPMPO-OOH spin-adduct formation with an IC_50_= 120 + 25 μM (Figure 2A). These results show NS1 inhibition of NOX (NOX2) activity at the cell membrane [27, 28].

Taking together, the kinetics of NOX levels changes induced by NS1 are consistent with a marked decrease of ROS and cell viability observed in A375 cells and in the other melanoma cells (Figures 1C–1E). Moreover, NS1 did neither inhibit normal melanocytes growth nor modify the levels of eNOS and NOX1, NOX2, NOX4 present in these cells, in contrast with DPI (Figure 1F).

The NOX proteins (NOX1, NOX2, NOX4) participate as integral signals associated with the unfolded protein response [28]. Stresses that perturb the redox status of the ER lumen, alter ER calcium levels, result in the accumulation of dysfunctional proteins in the ER, which initiates ER stress and the unfolded protein response (UPR) signalling [29]. We thus investigate whether NS1 may induce an ER stress.

### Early ER stress induced by NS1

We investigated the kinetics of several factors associated with ER stress in the presence of NS1 or DPI or L-NAME (Figure 2B and 2C). At early times of NS1 treatment (1-10h), increased expression of inositol-requiring enzyme 1α (Ire-1α) may promote ER Ca^2+^ release under ER stress. This Ca^2+^ release from the ER could be facilitated by the decrease of calnexin, a Ca^2+^-binding ER chaperone, clearly seen in A375 and SK-Mel 28 cells [28, 30]. The hypothesis of Ca^2+^ being released was ascertained by following up the fluorescence of Fluo-4 direct calcium reagent as a function of time (Figure 2D); a significant fluorescence increase was observed as early as 1 hour after NS1 treatment. Accordingly, an early increase in Ca^2+^ calmoduline- dependent kinase kinase II (CamKK2) phosphorylation was also observed, both calcium release and CamKK2 inducing autophagy (see below) [31]. Interestingly, only L-NAME treatment mimicked the increase of P-CamKK2 levels induced by NS1, suggesting an NO-related effect of NS1. The calcium efflux may trigger an ER stress. The hypothesis of an ER stress being induced was also supported by the increased expressions after 24 h of PERK, an ER stress sensor, of Bip, an ER-resident chaperone relying on energy exchange from the mitochondria from ATP or glycolysis and of CHOP, a transcription factor being induced in response to ER stress and involved in the induction of autophagy and apoptosis. Bip expression also increased after NS1 treatment in other melanoma SK-Mel28 and 1205 Lu (Figure 2C). Bip increase was also observed under DPI treatment, but Bip level was unaffected by L-NAME, rather suggesting a ROS-related effect of NS1 on Bip. In addition, NS1 rapidly increased the expression of Ero-1Lα, a protein associated with protein disulphide isomerase (PDI) involved in oxidative protein folding, which can produce ROS and impact mitochondrial function.

To test the potential link between calcium release and increase of ROS induced by NS1, we used the anti-oxidant N-acetylcysteine (NAC) and specific inhibitors of redox enzymes (Supplementary Figures S1 and S2). NAC treatment avoided both early calcium release and increase of ROS and NOX1/2, NAC linking early time modification of the calcium-calmodulin homeostasis with a redox stress and ER stress. We then used a panel of inhibitors. Clotrimazole was used as a potent inhibitor of calmodulin and calcium-dependent ion channel(s) that depleted calcium stores of cells exposed to clotrimazole without replenishment by normal cell mechanisms; this antifungal drug is also an inhibitor of the cytochrome P450 superfamily [32, 33]. Clotrimazole treatment increased Ire-1α and CHOP levels, further enhanced in the presence of NS1, while eNOS and calnexin expression decreased. Clotrimazole thus exacerbated the effect of NS1 on calcium-dependent ER stress and avoided modulation of ROS at 6h and 24 h. Rotenone increased ROS levels by uncoupling the mitochondrial complex 1 with overall increase of NOX1. Rotenone combined with NS1 treatment increased the ER stress, shown by Chop and calnexin levels. The PDI inhibitor strongly decreased overall ROS and NOX4 levels, with a marked decrease of the ER stress induced by NS1 at 24H shown by Ire-1α and p-CamKK2 levels. Allopurinol had only small effects on ROS, suggesting that xanthine oxidase is not a major source of ROS and ER stress. VAS2870 inhibited NOX4 and NOX2 [34] and mimicked NS1-induced increase of PERK after 24 h. Altogether, the calcium release mediated by NS1 triggers an increase of ROS and an ER stress, further enhanced by clotrimazole but inhibited by NAC.

To explore the links between cell viability and ER stress, we used silencing RNAs (Figure 2E). Silencing of Chop or Ire-1α antagonized NS1-induced cell viability decrease. These data link cell viability and ER stress.

Taken together, NS1 established an ER stress in melanoma cells that may be triggered by an early Ca^2+^ efflux, which decreased the cell viability.

### Induction of autophagy

**Figure 3:**
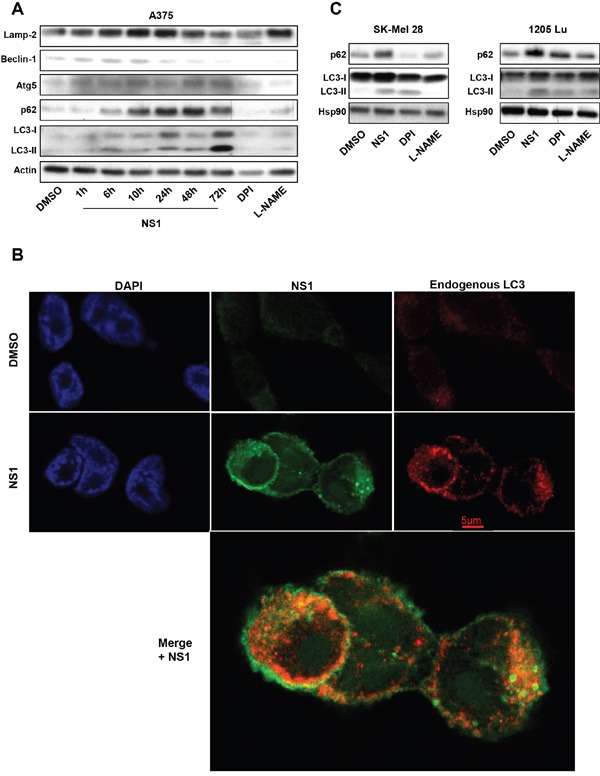
Effect of NS1 on autophagy: A375 melanoma cells **A.** or other melanoma cells **B.** were treated by 30μM NS1 or 30μM DPI or 100 μM L-NAME at indicated time. Cells were lysed and analyzed by western blotting using the indicated antibodies. HSP90 was used as a loading control. One representative experiment of three is shown. **C.** Immunofluorescence pictures of A375 melanoma cells treated with DSMO or 30μM NS1. LC3 was labeled with a secondary red antibody, NS1 was visualized after an excitation at 480nm (green) and DNA was visualized with DAPI (blue). Note the close localization between NS1 and LC3 seen by yellow/orange puncta clearly seen in the enlarged merged view.

The early calcium release (Figure 2D) could trigger autophagy [35, 36]. Autophagy is an evolutionarily conserved process by which damaged organelles and unneeded proteins are degraded by lysosomes to maintain intracellular homeostasis and to recycle cellular nutrients. Several autophagy-associated factors at early stages (Figure 3A) such as Lamp2, Beclin-1, Atg5 and p62 showed a time-dependant increase in their expression in response to NS1. The formation of LC3-II associated with autophagasome at the expense of the cytoplasmic LC3-I form started at 6H after NS1 treatment and peaked at 24 hours and above. We studied LC3 expression by immunofluorescence. We found that NS1 induces an increase of LC3 staining as well as LC3 puncta. Close localization of NS1 and LC3 was clearly observed in A375 cells by yellow/orange points. The punctuated form of LC3 strongly suggested that this close localization took place in autophagosomes (Figure 3B). Figure 3C shows that NS1 affected the autophagic markers LC3 and p62 in a similar way in all tested melanoma cells. In contrast, DPI and L-NAME affected p62 and LC3 levels quite differently in the different melanoma cells.

**Figure 4:**
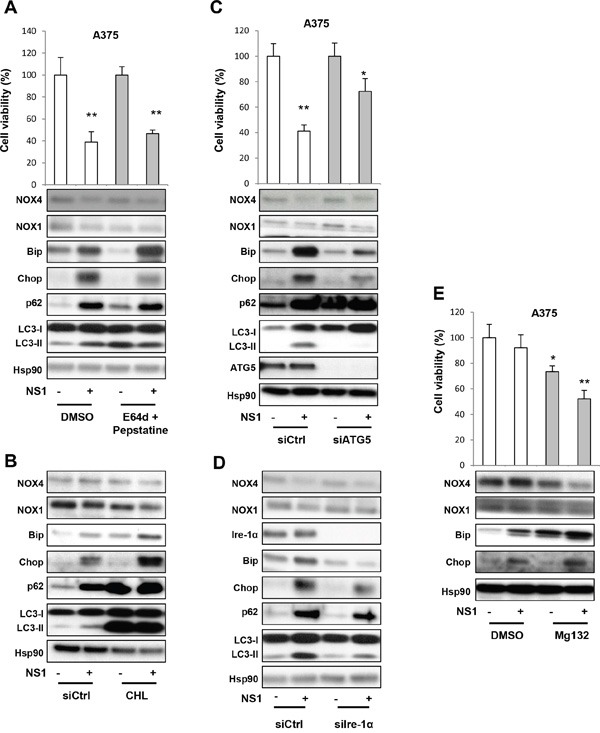
Evaluation of the autophagy in response to NS1 treatment using siRNAs and drugs **A.** A375 cells were treated with E64d + pepstatin (10μg/ml each) after 72 hours, and their viability was estimated by trypan blue staining. Analysis of different factors by WB is shown below. **B.** A375 cells were treated with chloroquine (100 μM) after 24 hours, and evaluated as in A. **C.** A375 melanoma cells were transfected with a siRNA against ATG5 (siATG5) or a siRNA control (siCtrl), treated as in A. **D.** A375 melanoma cells were transfected with a siRNA against Ire-1α (siIre1α) or a siRNA control (siCtrl), treated as in A. **E.** A375 melanoma cells were treated with a proteasome inhibitor Mg132 (10 μM) after 16 hours, and their viability estimated by trypan blue staining. The short treatment duration with chloroquine and Mg132 avoided their known cell toxicity after long exposure. In parallel, cells were lysed and analyzed by western blotting using the indicated antibodies. HSP90 was used as a loading control. One representative experiment of three is shown. The results are expressed as percentages of the control. Data are mean ± SD of three independent experiments performed in triplicate.

To get an insight on the mechanism associated with p62 accumulation, we first tested the autophagic flux with drugs, E64d and pepstatin, which are two lysosome drugs that inhibit the fusion of autophagosomes with lysosomes to form autophagolysosomes (Figure 4A). This treatment did not modify the cell viability decrease, Bip or Chop or p62 increase and LC3-II formation induced by NS1. Thus, NS1 modulates ER stress and autophagy at a step unaffected by these drugs.

To additionally test the effect of NS1 on autophagic flux, we used chloroquine (CHL) that impairs lysosomal acidification and block turnover of LC3-II to inhibit the flux immediately preceding the lysosomal degradation step. As expected, chloroquine induced LC3-II formation and p62 accumulation, with no further modifications of additional NS1 treatment (Figure 4B). However, combination of chloroquine and NS1 increased the levels of the ER stress markers Bip and Chop and further decreased NOX1 and NOX4 levels.

We then used silencing RNA directed against the important autophagic factor Atg5, which block early steps prior to autophagosome formation. SiAtg5 reduced the decrease of cell viability induced by NS1 compared with siRNA control (Figure 4C). SiAtg5 abolished LC3-II activation as did silencing RNA against the ER stress factors, siIre1-α and siCHOP (Figure 4D and Supplementary Figure S3). Moreover, siIre1-α and siCHOP reduced p62 levels, suggesting that there is a feedback between ER stress and autophagy at various levels.

Since the unfolded protein response interacts in a coordinated manner with the ubiquitin–proteasome system to alleviate protein misfolding and its cellular consequences, we finally tested the effect of a proteasome inhibitor: Mg132. Figure 4E shows that Mg132 alone decreased the cell viability after 8 hours of treatment. Co-treatment of Mg132 with NS1 synergized this loss of viability. This synergetic effect was also seen on the increase of ER stress markers as Bip and CHOP and decrease of NOX4 and NOX1 levels.

Taken together, NS1 drove autophagy at early steps leading to autophagosome formation as probed by siAtg5, in agreement with NS1 co-localizing with LC3 puncta. NS1did not affect later steps of fusion with lysosomes as probed by E64D and pepstatine. NS1-induced p62 accumulation suggests that NS1 is inhibiting selective autophagy at a late stage.

### NS1-induced cell death by apoptosis

**Figure 5:**
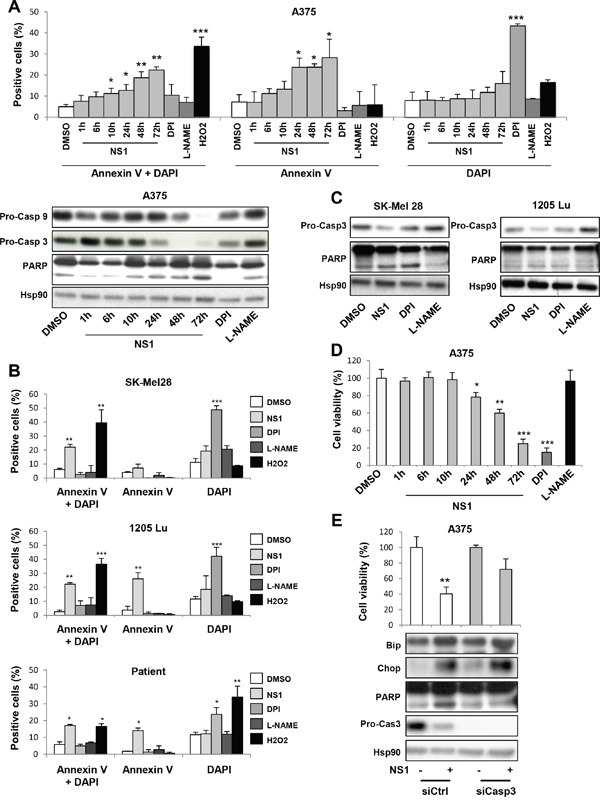
Evidence for apoptosis of melanoma cells treated by 30 μM NS1 A375 melanoma cells **A-B.** or other melanoma cells **C, D.** were treated by 30μM NS1 or 30μM DPI or 100 μM L-NAME or H2O2 at the indicated times. Cells were detached and their DNA contents were measured by flow cytometry; representative raw data of annexin V, DAPI and a double labeling are shown in panel A and B. In parallel, lysates were analyzed by western blot using the indicated antibodies (A lower part) and C). HSP90 was used as a loading control. **E.** A375 melanoma cells were transfected with a siRNA against caspase 3 (siCasp3) or a siRNA control (siCtrl), treated as in Figure 2E. The results are expressed as percentages of the control. Data are mean ± SD of three independent experiments performed in triplicate. One representative experiment out of three is shown.

Autophagy can promote cell death or protects cells and contributes to drug resistance. We investigated if NS1 induced cell death by apoptosis. Apoptosis was quantified by the Annexin V to DAPI quantification by FACS analysis (Figure 5A and 5B). In all melanoma cell lines including melanoma cells from a patient, the percentage of Annexin V- DAPI increased by 2 to 5 folds after NS1 treatment, strongly suggesting cell death was associated with apoptosis 72H after NS1 treatment. Apoptosis shown by the cleavage of PARP and the decrease of pro-caspase 9 and pro-caspase 3 was established in a progressive manner after NS1 addition (Figure 5A), observed from 24 hours, although a strong increase of Annexin V-DAPI started from 48h after NS1 treatment. NS1 strongly reduced A375 cell viability in a time-dependent manner (Figure 5D), reaching only 25% survival after 72h of treatment. Accordingly, the viability of melanoma cells freshly isolated from a patient at 72h with a wild-type BRAF and NRAS status also largely decreased upon NS1 treatment (Figure 1A). NS1 increased apoptosis and decreased the viability of the other melanoma cells, SK Mel28, which carried mutations of p53 and of BRAF, 1205 Lu cells mutated in BRAF and PTEN (Figure 5C). Collectively, NS1 treatment strongly reduced the viability of melanoma cells independently of their mutational status. NS1 triggered autophagy, the build-up of p62 suggesting that autophagy is incomplete (Figure 4) and induced apoptosis (Figure 5). SiCasp3 partly restored the cell viability loss induced by NS1, showing that apoptosis is responsible of a death induced by NS1 (Figure 5E). Further, Supplementary Figure S4 indicates that ER stress is downstream autophagy and apoptosis and that autophagy is downstream apoptosis.

### Signalling pathways involved in ER stress, autophagy and cell death induced by NS1

**Figure 6:**
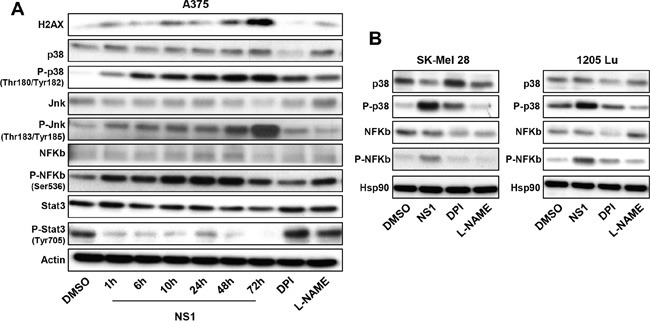
Effect of NS1 on signalling pathways A375 melanoma cells **A.** or other melanoma cells **B.** were treated by 30μM NS1 or 30μM DPI or 100 μM L-NAME at indicated time. Cells were lysed and analyzed by western blotting using the indicated antibodies. HSP90 was used as a loading control. One representative experiment of three is shown.

ER stress induced by NS1 treatment was concomitant with p38 and NF-κB activations shown by the increased phosphorylation of both proteins on western blot (Figure 6A), a process that may arise from PERK (and Ire-1α) activation by oxidative/ER stress as suggested from Figure 2. These activated pathways in A375 were also activated in other melanoma cells, SK-Mel28 and 1205Lu (Figure 6B). The early calcium release (Figure 2D) was combined with JNK activation as observed by increase of JNK phosphorylation (Figure 6A). Evidence for cell damage signalling was given by the enhancement of the histone H2a variant H2AX levels associated with double-stranded breaks after 48-72 h (Figure 6A), processes triggering autophagy [35, 36]. Remarkably, a strong reduction of phosphorylated STAT3 levels was clearly observed at early times (Figure 6A).

## DISCUSSION

### NS1 induced an ER stress

NS1 established an ER stress mainly due to a large release of calcium. The increased expression of Ire-1α may promote ER Ca^2+^ efflux, facilitated by the decrease of calnexin, a Ca^2+^-binding ER chaperone clamping on the activity of the SERCA pump [30]. This Ca^2+^ release to the cytosol is consistent with JNK/c-Jun activation. Ire-1α, CHOP and Bip and later increase of PERK expression levels were clearly enhanced.

We suggest that NS1 directly modulate (a) calcium-dependent channel(s), due to its shared nucleotide motif with both NADP and nicotinic acid adenine dinucleotide phosphate (NAADP) [37]. The ryadonine receptor is a calcium release channel that binds pyridine nucleotides as NADP+ and NAADP due to the structural homology of its N-terminal domain with oxido-reductase –like domain [38]. The two-pore channel is involved in binding NAADP and in releasing calcium from ER stores [39, 40]. We further test this hypothesis by using clotrimazole, an antifungal drug which blocks calcium-activated K^+^ channels with an IC50 value of 0. 92 μM [33] and calcium-permeable ion channels TRPM2 [41], disrupts ER calcium homeostasis, inducing ER stress. Indeed, NS1 synergized with clotrimazole by increasing the calcium-dependent ER stress, suggesting NS1 affects such a calcium-dependent channel. We cannot exclude that some effects of clotrimazole and NS1 are related to their inhibition of P450/P450 reductase uncoupling [19]. The inhibition by NAC of calcium and ROS increase likely indicates a redox regulation of this channel [42]. However, under conditions of increased calcium efflux (clotrimazole) or enhanced ROS production from the mitochondria (rotenone), the redox modulation of the channel cannot take place anymore as suggested by the loss of NS1 modulation on ROS levels. Accordingly, a marked decrease of ER stress at 24H was observed by inhibiting PDI, a protein that couples with NOX to allow oxidative folding in the ER [43, 44]. Direct inhibition of NOX4 and NOX2 by VASP2870 mimicked NS1-induced ER stress shown by PERK and CHOP levels.

In addition, NS1 inhibition of eNOS activity may affect Ca^2+^-dependent channel(s) in close proximity with the caveolae by an indirect effect. The calcium release may result in NOX1/2 activation [45]. NOX5 calcium-dependent activity being increased by eNOS inhibition is unlikely (Supplementary Figure S5) [46, 47].

Altogether, NS1 likely directly or indirectly affects a calcium-dependent, redox-sensitive ion channel modulated by NOX [42]. More work is needed for this ion channel identification.

### Induction of incomplete autophagy in melanoma cells with different mutational status linked with ROS inhibition

As a response to ER stress, the cells responded by coordinating activation of the canonical NF-κB pathway and the autophagy machinery [48]. Increased expressions of Lamp-2, Atg5, Beclin-1 LC3-II and p62 were consistent with autophagy being induced (Figure 3). Importantly, NS1 localized close to LC3 in the autophagosome, suggesting that NS1 may inhibit ROS formed by NOX(s) in the autophagasome. NOX1 and NOX2 have been associated with autophagy, since ROS production is required for phagosome function [49, 50]. Very similar pathways as NF-κB signalling associated with triggering autophagy were observed in all melanoma cells tested. Interestingly, we note that 1205 Lu cells were more sensitive to NS1 treatment than A375 cells: these cells presented a more pronounced decrease in cell viability and enhanced apoptosis, ROS inhibition was more efficient; p62 accumulation was observed upon NS1 or DPI treatment, while it was not the case for other cell lines. 1205Lu carry the BRAF mutation and PTEN loss while A375 are mutated only in BRAF [51]. The p53 level was not significantly modified by NS1 treatment (data not shown) and is not likely to be involved in triggering autophagy.Indeed, we tested SK-Mel28 cells in which p53 is mutated and still observed the same effect of NS1 than in A375 cells. The same ER stress, autophagy block and apoptosis were observed with cells from a patient with wild type BRAF and NRAS. Collectively, the data suggest that NS1 could induce cell death in melanoma carrying various mutations as shown by the efficacy of NS1 treatment on 1205Lu cells. Of great interest, NS1 treatment was also effective in melanoma cells resistant to treatment with a BRAF inhibitor as suggested by preliminary results (Supplementary Figure S6). ER stress associated with incomplete autophagy could be the reason for the effectiveness of NS1 observed in all tested metastatic cell lines and cells isolated from seven patients and in PLX-resistant cells but not in melanocytes (Figures 1–3 and Supplementary Figures S5, S6) [52].

**Figure 7:**
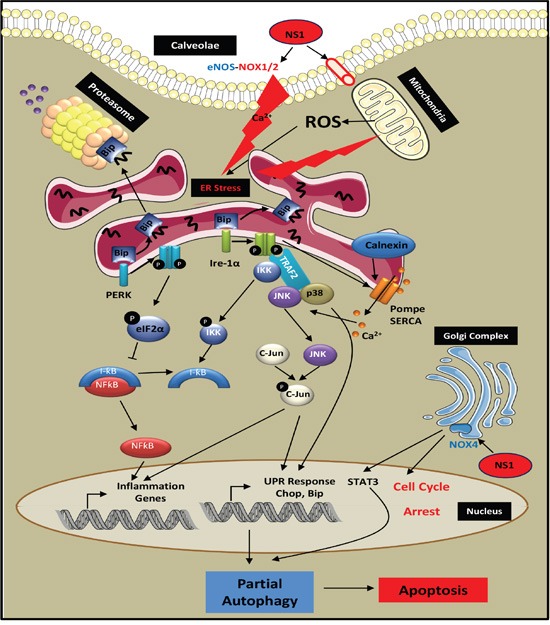
Scheme of potential mechanisms for NS1-induced melanoma cell death By a direct effect of NS1 on a calcium redox-dependent ion channel or by an indirect effect of NS1 mediated by eNOS inhibition in close proximity to caveolae, NS1 induced an early ER stress arising from a release of calcium sensed at the ER by Ire-1α, calnexin, BIP and PERK that triggered JNK, p38 and NF-kB signalling. NS1 inhibition of angiogenesis and cell cycle blockade via NOX4 decrease could be associated with down-regulation of STAT3 signalling. These redox, calcium and metabolic stresses triggered autophagy, evidenced by an increased formation of LC3-II in autophagosomes that co-localized with NS1. NS1 inhibition of ROS formed by NOX1 likely blocked autophagy relying on ROS for further fusion of autophagosomes with lysosomes required for autophagy completion. This partial autophagy is consistent with the build-up of p62 selective autophagy cargo, accumulation of DNA damage and specific effects of siRNAs on the autophagy and cell survival. This autophagy blockade resulted in cell death by apoptosis in melanoma cells carrying or not different mutational statuses.

NS1 inhibition of NOX1 is the likely cause of autophagy blockade, shown by the accumulation of p62, a key cargo adaptator for the degradation of ubiquinated substrates [53]. The overall ROS decrease after 24 hours (Figure 1) is consistent with NOX1, NOX2 and NOX4 decrease (Figures 1C, 2A). IC_50_ for NS1 inhibition of NOX2 is 120 ± 25 μM using EPR detection relying on spin-trapping of superoxide ions. Lower IC50 values for NS1 inhibition of NOX2 using fluorescence changes are consistent with the inhibition of ROS by 30 μM NS1 (unpublished data). NS1-induced inhibition of NOX2 by blockade of the NADPH site likely applies to other NOX isoforms. NS1 also inhibits eNOS [19, 24]. Both eNOS and NOX1/2 are located within membrane rafts called caveolae, and associates with caveoline-1 (cav-1) a protein of caveaolae [54] (Figure 7). Local cross-talk exists between Cav-1, overexpressed in melanoma, and NOX1, modulating NOX1 activity [55]. The increased level of eNOS expression may reflect this cross-talk with NOX1 or with NOX4 as suggested [24]. NS1 inhibition of NOX4 is likely linked with its anti-angiogenic effect and its induction of cell cycle blockade [24]. Hyperactivation of the STAT3 pathway, a downstream target of ROS-activated JAK2 kinase signalling, is intimately linked with melanoma development and the acquisition of tumor invasiveness [56]. NS1 inhibition of STAT3 possibly linked with NOX4 inhibition may reduce cell viability and invasive properties in naïve and resistant melanoma [57]. Collectively, our results show that NS1 triggers an incomplete autophagy resulting in cell death, in agreement with p62 accumulation, siAtg5 reversing NS1-induced decrease of cell survival and the lack of effects of E64d + pepstatin acting on later stages of autophagy. Figure 7 proposes a mechanism by which NS1 could induce melanoma cell death.

To investigate a potential link between patient survival and eNOS and NOX levels, we used bibliographic data of a cohort of patients followed up for two years after excision of melanoma metastatic lesions [58]. In this cohort, patients with a low eNOS mRNA expression showed an overall lower survival (Supplementary Figure S5). Furthermore, high grade of NOX1 and NOX4 mRNA levels was linked with lower patient survival [10, 25]. Related caveolin-1 levels in serum of melanoma patients were found to be of clinical significance [59]. Collectively, these data suggest a role of eNOS, NOX1 and NOX4 in metastatic melanoma. In this context, NS1 treatment induced both the increase in eNOS and decrease of NOX1 and NOX4 suggested to be linked with better patient survival [58]; neither DPI nor L-NAME displayed such a trend.

### Perspectives

Here we hypothesize that, by regulating more than one signaling mediators as NO and ROS, small targeted-molecules like NS1 may alter redox and NADPH-dependent fluxes between the tumor and its micro-environment and possibly leading to a “normalization” [12, 60] and reduction of angiogenesis/metastasis. Tumor consumption of lactate, linked with NADPH via the tricarboxylic acid cycle, is required to maintain growth-autophagic flux. To further explore metabolic changes induced by NS1 in melanoma cells, we determined lactate levels (Supplementary Figure S7). NS1 decreased lactate levels produced by aerobic glycolysis in all melanoma cells and siAtg5-induced block of autophagy amplified this effect of NS1. Taking together, NS1 may normalize the tumor micro-environment by reducing redox and metabolic fluxes. NS1 ability to induce ER stress and a partial autophagy leading to apoptosis of metastatic melanoma cells with various mutational statuses combined with its unique imaging of specific targets is of great value for future therapeutic perspectives.

To conclude, we demonstrate that our compound NS1 induces melanoma cell death through incomplete autophagic and apoptotic mechanisms initiated by ER stress. Taking account the drastic effect of NS1 on melanoma cells combined with a lack of apparent toxicity to normal cells, this study provides compelling data to support the idea that an NADPH analogue as NS1 could be useful either alone or as an adjuvant therapy in melanoma treatment.

## EXPERIMENTAL PROCEDURES

### Reagents

NS1 was synthesized at a larger scale (1 g) by a private company, GreenPharma (Orléans, France) using a previously described protocol [19] and purified by chromatography to a single peak at (M-H)- = 653 in mass spectrometry; the compound had a small enrichment of the 3′-PO_4_H_2_ over the 2' regioisomer compared to the previous 1/1 ratio. NS1 was dissolved in DMSO at a final concentration of 10 mM, protected from light, aliquoted and stored at −20°C. Catalase, calmodulin (CaM), MgCl_2_, KCl, L-N^G^-nitroarginine methyl ester (L-NAME), Hepes, E64d (10μg/mL) inhibitor of autophagy and protease, Pepstatin (10 μg/mL), rotenone, 16F6, allopurinol and VAS2870 were from Sigma-Aldrich (Saint Quentin Fallavier, France). DMEM culture media and endotoxin-low fetal calf serum (FCS), CellROX^®^ Deep Red Reagent were purchased from Invitrogen (Pontoise, France). 5-Diethoxyhosphoryl-5-methyl-pyrroline N-oxide (DEPMPO) was from Radical Vision (Marseille, France). NOX 1 and NOX4 antibodies were purchased from Abcam (Paris, France), antibodies against Hsp90, p62 were from Santa Cruz Biotechnology (TEBU, Le Perray en Yvelines, France). The polyclonal pro-caspase 9 was from Cell signaling Technology (Ozyme, Saint-Quentin-en-Yveline, France). Antibodies against PARP, Caspase 3 cleaved, JNK, Phospho-JNK, p38, Phospho-p38, Ire-1α, Ero-1Lα, PERK, Bip, Calnexin, LC3, NF-κb, Phospho-NF-κb, Stat3, Phospho-Stat3, Beclin, Atg5, Lamp-2, anti-eNOS and anti-Pro-Caspase 3 were from BD Bioscience (Pont de Claix, France) and anti-H2AX from Merck Millipore (Fontenay-sous-Bois, France).

### Cell culture and treatments

Human A375 (CRL-1619) and SK-Mel 28 melanoma cells were purchased from American Tissue Culture Collection (Molsheim, France) and grown in RPMI medium supplemented with 10% FCS at 37°C in a humidified atmosphere containing 5% CO_2_. 1205Lu cells were grown in DMEM with 10% of FCS. Epidermal cell suspensions were obtained from foreskins of Caucasian children by overnight digestion in phosphate-buffered saline containing 0.5% dispase grade II at 4°C, followed by a 20 min digestion with 0.05%trypsin–0.02% EDTA in phosphate-buffered saline (V/V) at 37°C. Normal human melanocytes were grown in MCDB153 medium supplemented with 2% FCS, 0.4mg/ml hydrocortisone, 5 mg/ml insulin, 16 nM phorbol-12 myristate 13-acetate, 1 ng/ml basic fibroblast growth factor, 10 mg/ml bovine pituitary extract and penicillin/streptomycin (100 U/ml/50 mg/ml. Melanoma cells isolated from patients were prepared as described [24]. For each experiment, cells were starved without 1% FCS in appropriate medium during 14 h before drug stimulation. Cultured mouse macrophage cell line RAW 264.7 cells (Sigma Aldrich) were grown to ~90% confluence in 75 cm^2^ flasks containing 15 ml DMEM growth medium supplemented with 5% FCS, 10 U/mL penicillin and 100 μg/mL streptomycin under a 5% CO_2_ atmosphere at 37°C.

### Transient transfection of small interfering RNA

Briefly, a single pulse of 50 nmol/L of small interfering RNA (siRNA) was administrated to the cells at 60% confluence by transfection with 5 μL Lipofectamine RNAiMaX in Opti-MEM for 24h. The cells were starved and treated with NS1 (30μM) after 72h. SiAtg5 and siIre-1α were from GE healthcare (Aulnay sous Bois, France), siCasp3, siCHOP from life technologies (Thermo Fisher Scientific, Carlsbad, CA, USA) and siBip from Santa Cruz.

### Cell viability of MHN and melanoma cells

For the study of the effect of NS1 on NHM and various melanoma cells, viable cells were counted using trypan blue dye exclusion method as previously described [61].

### Fluorescence detection of ROS generation

CellROX^®^ Deep Red Reagent was used for oxidative stress detection (absorption and emission maxima at ~644/665 nm). A375 cells (1-1.5x10^5^ cells) plated in 12-well plates at ~85% confluence were pre-incubated with increasing concentrations of NS1 for 30 min at 37°C, and then further incubated with 2 μM CellROX^®^ Deep Red Reagent for 30 min. Tert-butyl hydroperoxide (TBHP) (Sigma Aldrich), an oxidative stress inducer, was used as a positive control. After centrifugation at 1,000 rpm for 5 min, the cell pellets were resuspended in PBS containing 30% of enzyme-free Cell Dissociation Buffer (Gibco^®^, Thermo Fisher Scientific, Carlsbad, CA, USA) and analysed by FACS Calibur flow cytometer (Becton-Dickinson, Pont de Claix, France). To address the modulation effect of NS1 on ROS formation, the MFI (Mean Fluorescence Intensity) related to fluorescence emission of the CellROX^®^ Deep Red Reagent, measured in the presence of a given concentration of NS1, was normalized by the MFI obtained in the absence of NS1 (*i.e.* CellROX^®^ Deep Red Reagent alone), giving the fluorescence enhancement factor, leading to a value of ROS positive cells in %.

### Superoxide anions formed by RAW 264.7 cells

Cultured RAW 264.7 cells were stimulated by PMA and superoxide anions generated by the NADPH oxidase activity were trapped with the cylic nitrone DEPMPO and measured by EPR detection of the DEPMPO-OOH spin-adduct [62]. Cells (~3.10^+6^ in 15 cm^2^ flask) were washed with fresh DMEM containing 5% FCS, incubated 20 min at 37°C in DMEM containing 10 μM PMA and 25, 50, 100 or 250 μM NS1, or 50 μM DPI. The medium was removed and the cells were washed twice with 5 mL PBS, detached, and centrifuged. The pellet was washed with 5 mL fresh DMEM containing 5% FCS, centrifuged, and washed again with 5 mL PBS. The cells were then re-suspended in 80 μL PBS containing 100 μM DTPA and 25 mM DEPMPO, and introduced into a Teflon capillary inserted into the shq001 cavity of a Bruker Elexsys 500 EPR spectrometer (Bruker, Wissembourg, France). Data accumulation started immediately. All measurements were carried out at 21°C. The following instrument settings were used: field modulation amplitude, 2 G; time constant, 40.96ms; conversion time, 40.96 ms; microwave power, 10 mW; field width, 120 G; center field, 3490 G; scan time, 41.94 s; number of scans, 16. DEPMPO-OOH spectrum (hyperfine splitting constants: A_N_, 13.4G, A_P_, 52.5 G, and A_H_ 11.9 G) was identified by comparison with incubations performed in the presence of xanthine/xanthine oxidase. The DEPMPO-OOH spin adduct slowly decomposed to the DEPMPO-OH spin adduct under our conditions (half-life of about 25 min) and the amounts of DEPMPO-OH spin adduct were neglected under our conditions. The changes in the amplitude of the first peak of the DEPMPO-OOH adduct as a function of time were used to quantify the amounts of superoxide generated in the experiments and the rates of these changes were plotted for 10 min. Control experiments were performed with cells non treated with PMA.

### Flow cytometry analysis

Cells exposed to NS1 or DPI or L-NAME were detached with Hqtase and stained with AnnexinV and with DAPI for apoptosis or DAPI for cell cycle. Apoptosis profiles and cell cycle was determined by flow cytometric analysis as described before [63]. Cell cycle and apoptosis profiles were collected using a FACScan instrument and analyzed with the CELLQUEST software (Becton-Dickinson).

### Western blots in A375 and other melanoma cells

Western blot analyses were performed as described [61].

### Immunofluorescence studies

A375 melanoma cells were grown on glass coverslips (100000 cells per point) in 6-well dishes and treated for 24h with 30μM of NS1 after 14h of starvation. Cells were then washed, fixed at room temperature for 20 min with 3, 7% of paraformaldehyde, and permeabilized by 2 min with phosphate-buffered saline 1% Triton before being exposed to an anti-LC3 antibody for overnight at 4°C. Cells were next incubated with Alexa Fluor^®^ 647 (life technologies) coupled anti-rabbit for 1h at room temperature and the cells were washed with phosphate-buffered saline. Finally, coverslips were mounted in moviol immunofluorescence mounting medium and examined with 60x objective using Nikon A1R.

### *In vitro* intracellular calcium assay

Fluo-4 direct calcium assay kit from Invitrogen was used for monitoring the intracellular calcium response to NS1 in A375 melanoma cells. A375 were cultured in a 96-well plate. After different times of treatment with NS1 30 μM, cells were incubated with Fluo-4 direct calcium reagent loading solution for 30 min. After incubation, the fluorescence signal was immediately measured using a fluorescence microplate reader at excitation and emission of 509 and 516 nm, respectively.

### Statistical analysis

All data were presented as means ± standard deviation (SD). Statistical analysis was performed using the Student t-test: * P < 0.05 versus control, ** P< 0.01 vs control, *** P < 0.001 deemed statistically significant.

## SUPPLEMENTARY DATA, SUPPLEMENTARY METHODS



## Abbreviations

CaM: calmodulin
DPI: diphenyleneiodonium
E64D, 2S, 3S-trans: Ethoxycarbonyloxirane-2-carbonyl)-L-leucine-(3-methylbutyl) amide (Calpain and cathepsin inhibitor)
DEPMPO: 5 diethoxyphosphoryl-5-methyl-1-pyrroline N-oxide
FCS: fetal calf serum
n-,i-and eNOS: neuronal, inducible and endothelial nitric oxide synthase, respectively
L-NAME: L-NG-nitroarginine methyl ester
NHM: normal human melanocytes
NOX: NADPH oxidase
PMA: phorbol 12-myristate 13-acetate
ROS: reactive oxygen species
TBHP: tert-butyl hydroxyperoxide.
