# Supplementary material for: Mechanism of melanoma cells selective apoptosis induced by a photoactive NADPH analogue
Source: Oncotarget. 2016 Oct 14;7(50):82804–19. doi: 10.18632/oncotarget.12651 (PMC5347734; doi:10.18632/oncotarget.12651)
Supplement: Supplementary file 1 [file oncotarget-07-82804-s001.pdf]

Mechanism of melanoma cells selective apoptosis induced by a photoactive NADPH analogue

SUPPLEMENTARY DATA

SUPPLEMENTARY METHODS

Lactate measurement in A375 melanoma cells: For experiments quantifying lactate consumption or production in A375 culture media, cells were incubated with NS1 30 µM or DPI 30 µM or L-NAME 100 µM. Medium lactate were quantified using the YSI 2900 Biochemistry Analyzer (YSI Life Sciences).

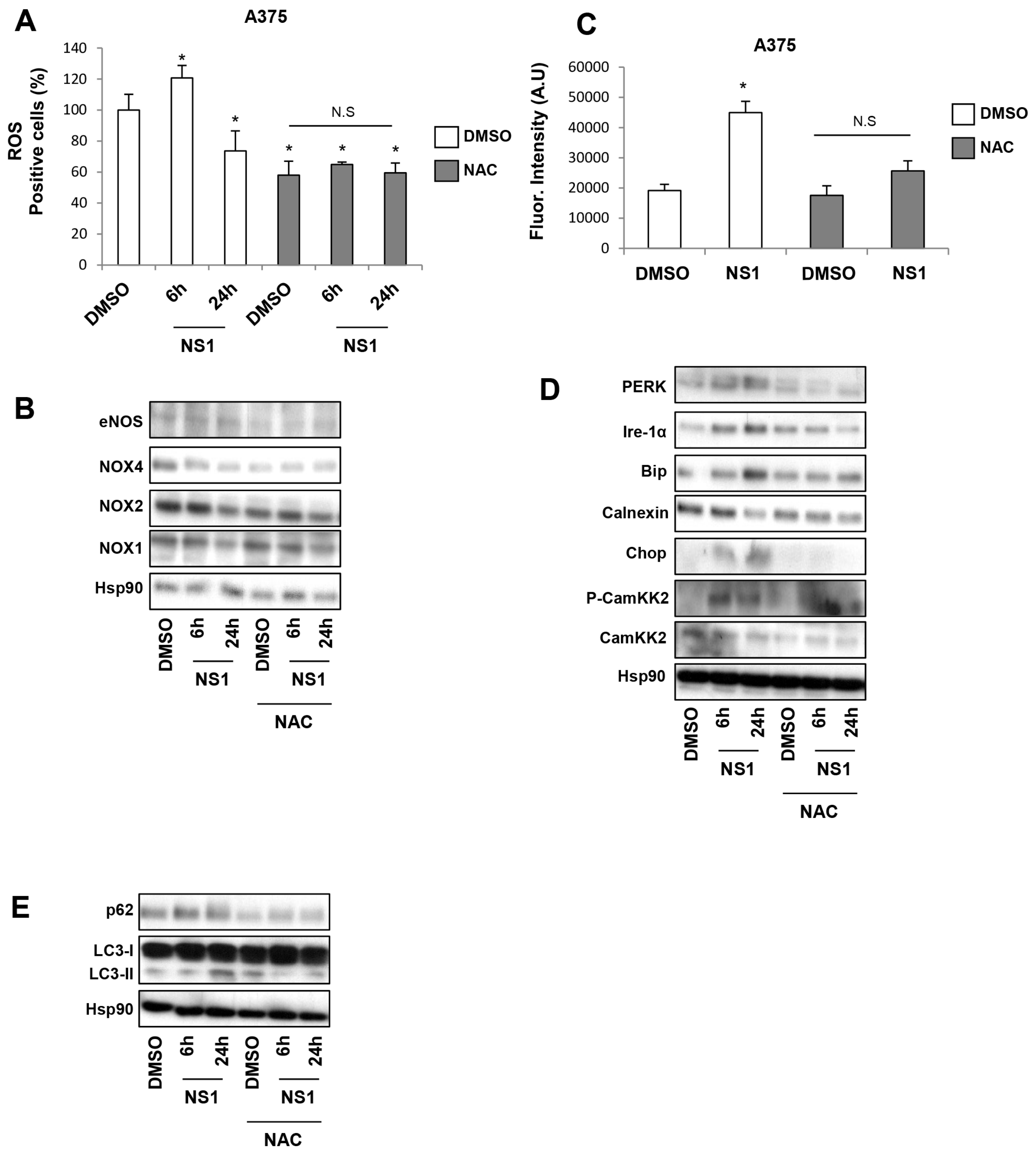

Supplementary Figure S1: Probing the early increase of calcium and ROS with N-acetyl cysteine (NAC); the cells were pre-treated with NAC used at a concentration of 5 mM during 2 hours before applying NS1 during 24 hours. Note that by applying NAC both early ROS (panels **A, B**) and calcium increase (panel **C**) were avoided, in agreement with decrease in ER stress markers PERK, Ire-1α, Bip, Chop and pCamKK2 (panel **D**) and autophagy factors p62 and LC3-II (panel **E**).

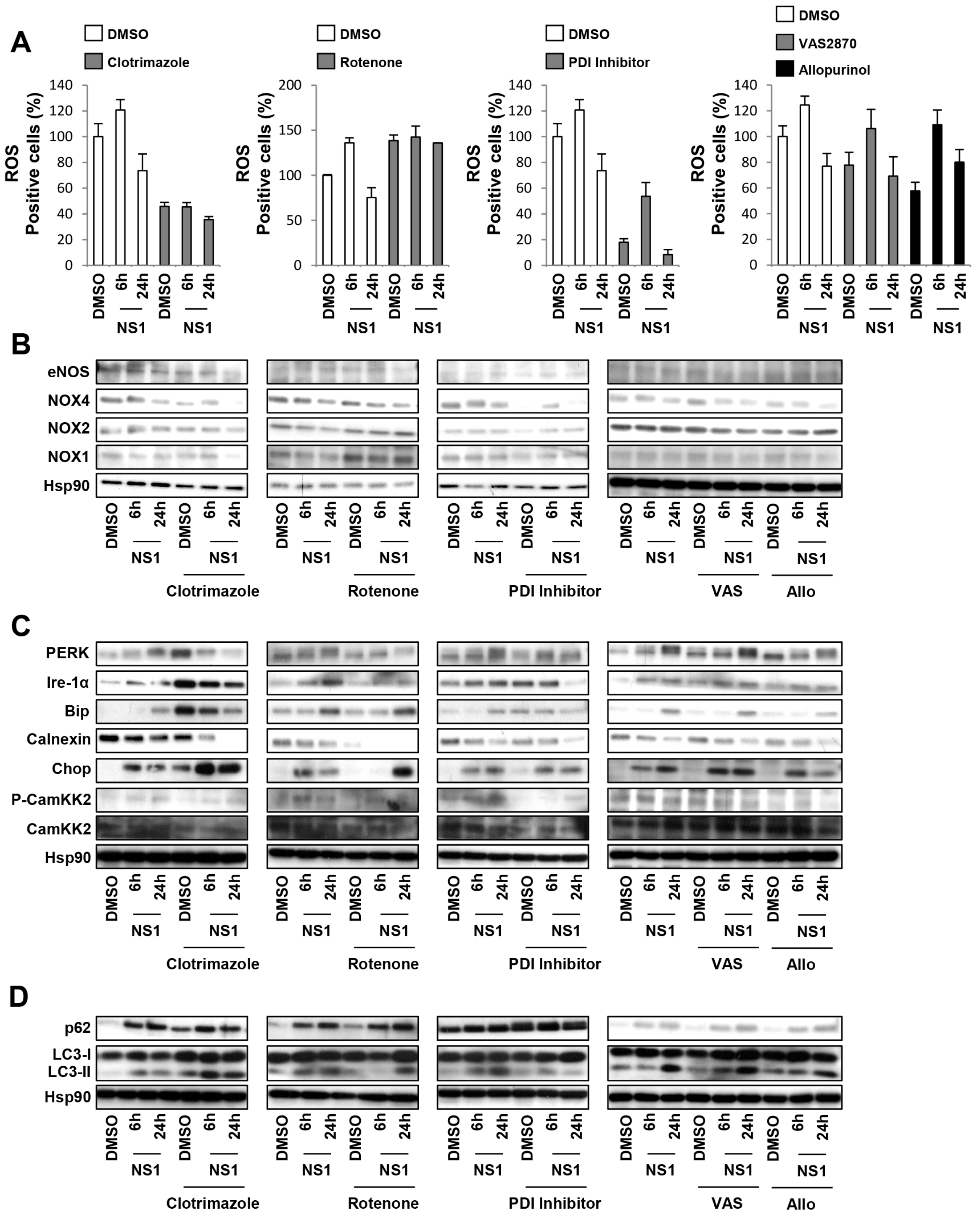

Supplementary Figure S2: A. Probing the source of early ROS increase, B. NOX change and C & D. ER stress and autophagy markers with inhibitors of selected redox enzymes: A: Clotrimazole 5µM, an inhibitor of calmodulin and calcium- dependent channels; rotenone (0.5 µM) mitochondrial chain inhibitor; 16F16 (4µM), PDI inhibitor; VAS2870 (10 µM), (general) NOX inhibitor; allopurinol (100 µM), xanthine oxidase inhibitor.

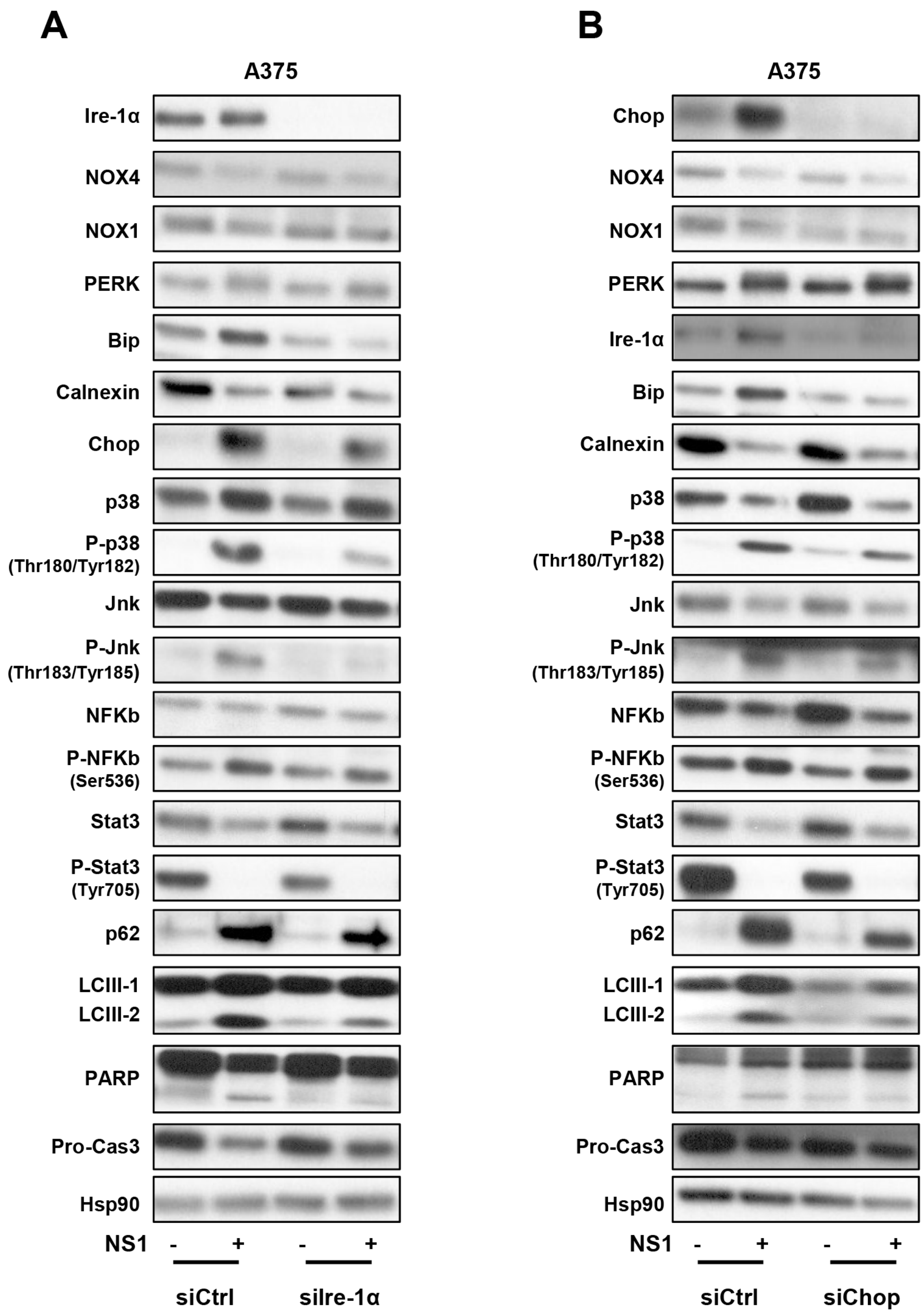

Supplementary Figure S3: Links between selected autophagy, ER stress and apotosis markers by silencing Chop, and Ire-1α and their effect on signalling.

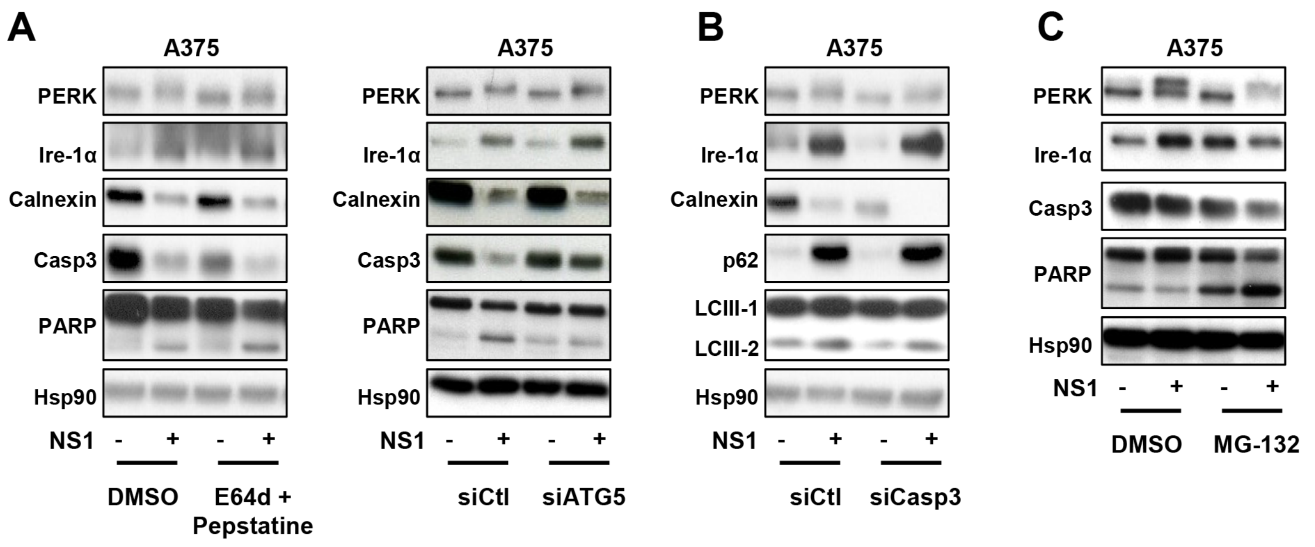

Supplementary Figure S4: Relationships between ER stress and autophagy probed by treatment with E64d + pepstatin or siATG5 A., and apoptosis probed by the use of siCaps3 B. and proteasome via the Mg132 proteasome inhibitor C.

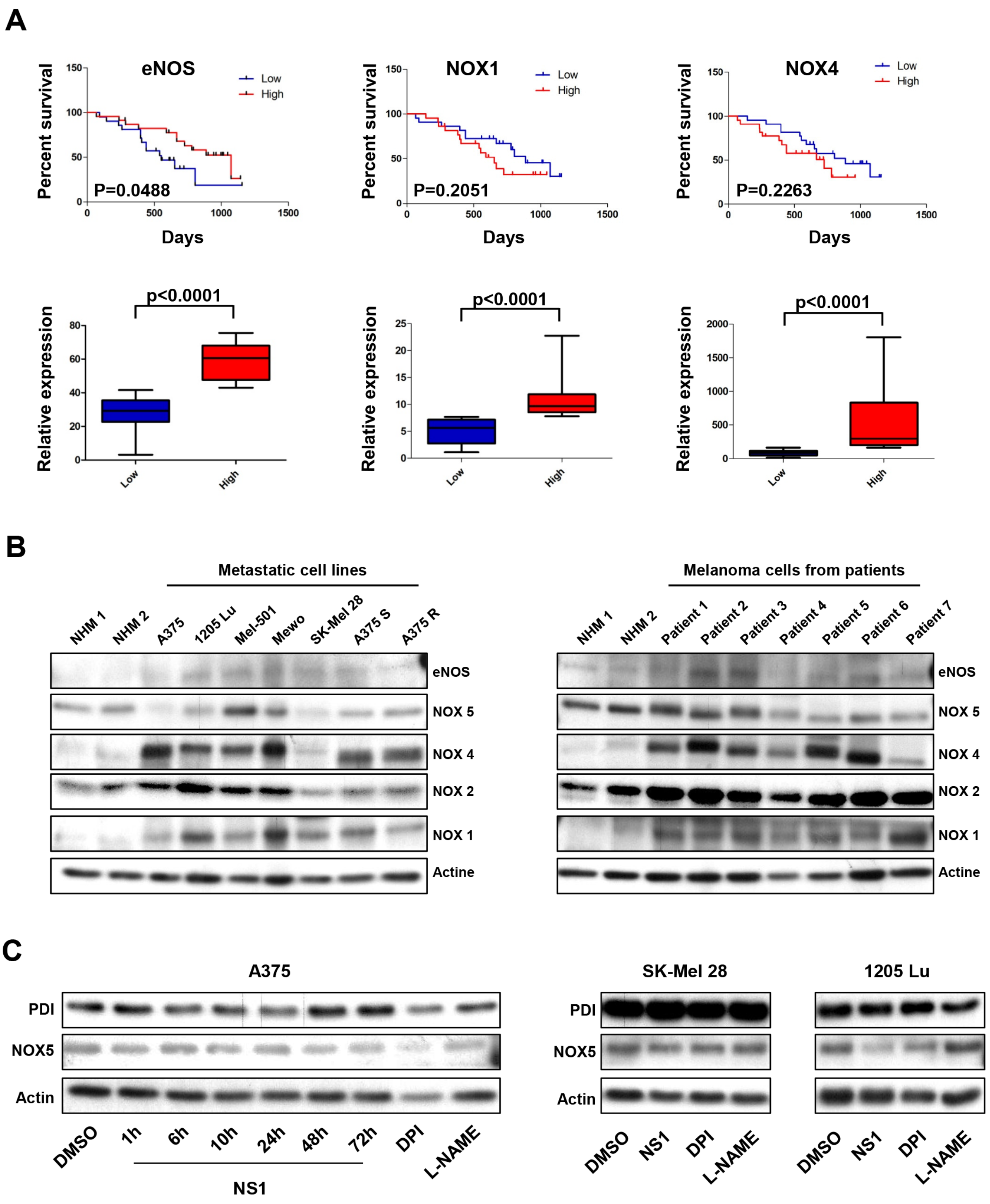

Supplementary Figure S5: A. Survival of melanoma patients with high and low (above or below the median, respectively) mRNA levels of eNOS, NOX1 and NOX4. Gene expression data of 44 metastatic melanoma tissues were used to define high and low expressor groups (boxplots) and to generate Kaplan-Meier curves. Values were normalized to GAPDH. **B, C.** Comparison of eNOS and NOX1, NOX2, NOX4 and NOX5 levels in metastatic cell lines and in cells from 4 healthy donors and from 7 patients (this study).

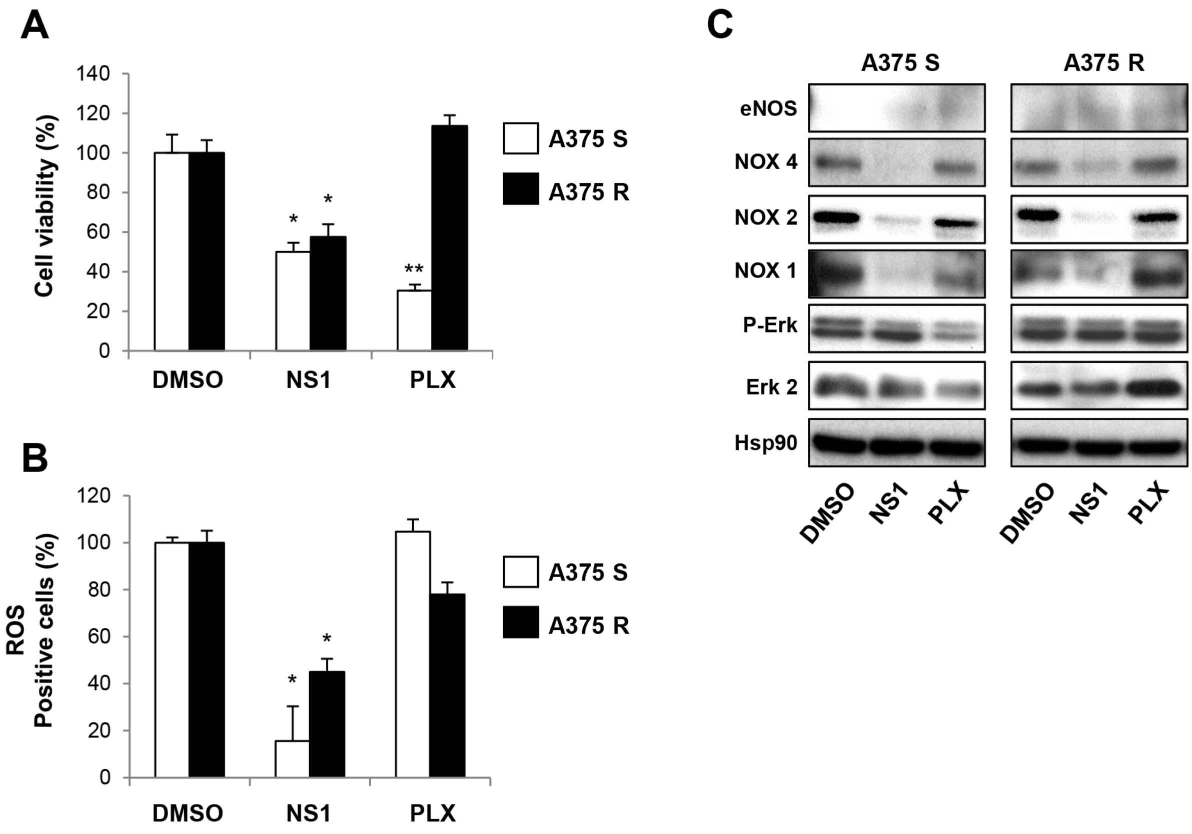

Supplementary Figure S6: Comparing the effect of NS1 treatment on sensitive and B-RAF resistant A375 cells: **A.** cell viability after 72 hours **B.** ROS levels **C.** in both naïve and resistant cells, NS1 decreased NOX1, NOX2 and NOX4 levels. NOX1 level was enhanced in PLX-4032 (5µM) treated resistant cells compared to sensitive cells.

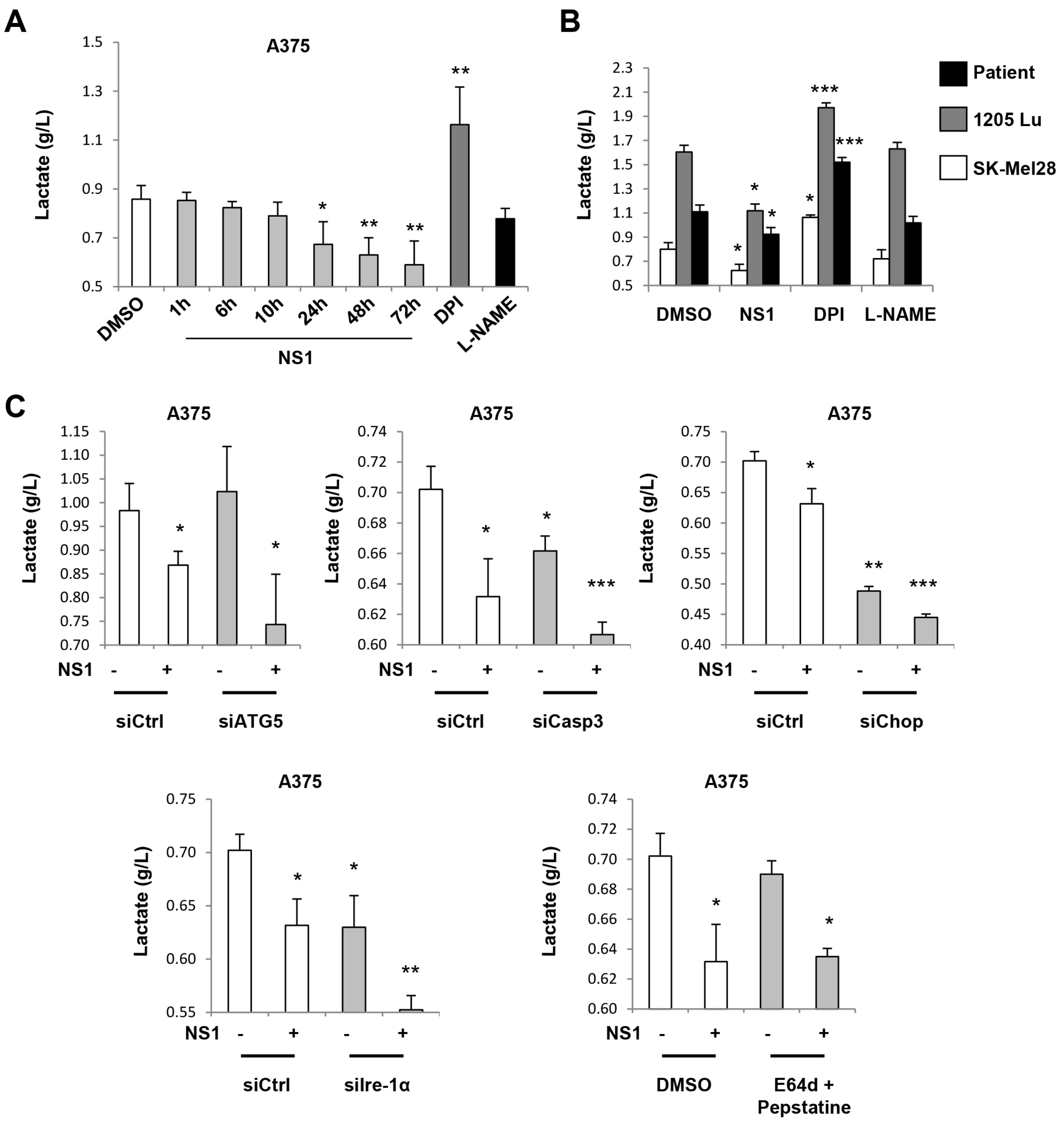

Supplementary Figure S7: Decrease of lactate levels following NS1 treatment: A. kinetics of lactate levels as a function of time in A375 cells; **B.** Lactate levels at 72H post-treatment with NS1 compared with either DPI or L-NAME treatment in various melanoma cells; **C.** Links between lactate metabolism and ER stress, autophagy and apoptosis via the effects of siRNAs: siAtg5, siCasp3 and siCHOP, siIre-1α and treatment with E64d and pepstatin on lactate levels in A375 cells.
